# Effective treatment of aggressive fibromatosis with celecoxib guided by genetic testing

**DOI:** 10.1080/15384047.2017.1373215

**Published:** 2017-09-07

**Authors:** Shanshan Yang, Xufu Wang, Haiping Jiang, Yongjie Wang, Zhuokun Li, Haijun Lu

**Affiliations:** a Department of Oncology, The Affiliated Hospital of Qingdao University, Qingdao, Shandong, China; b Department of Nuclear Medicine, The Affiliated Hospital of Qingdao University, Qingdao, Shandong, China; c Department of Thoracic Surgery, The Affiliated Hospital of Qingdao University, Qingdao, Shandong, China; d BGI-Qingdao Institute, Qingdao SINO-GERMAN Ecopark, Qingdao, Shandong, China; e BGI-Shenzhen, Shenzhen, Guangdong, China

**Keywords:** aggressive fibromatosis, celecoxib, CTNNB1gene mutations, genetic testing

## Abstract

Aggressive fibromatosis (AF) or desmoid tumors is an aggressive fibroblastic proliferation which is locally invasive but can not metastasize. The treatment of AF is challenging. Surgery was the main treatment modality for AF in the past, other strategies including radiotherapy, systemic therapies and wait-and-see policy. The use of non-steroidal anti-inflammatory drugs (NSAIDs) and targeted therapies has demonstrated good results. In the case report, a 39-year-old man presented with progressive chest wall pain. Computed tomography (CT) showed an approximately 46× 13 mm soft-tissue mass between the inside of the fifth and sixth rib on the right side. The entire mass was excised and an AF was confirmed based on histopathology. Four months after surgery, magnetic resonance imaging (MRI) showed a soft-tissue mass in surgical areas and biopsy confirmed local recurrence. Therefore, Tomotherapy was administered. However, two months later, an (18)F-fluorodeoxyglucose (FDG) Positron Emission Tomography combined with CT (PET-CT) revealed the presence of an FDG-avid mass in the area of local recurrence. Genetic testing reported the presence of a p.T41A mutations on the CTNNB1 gene, which predicted that he is sensitive to the COX-2 inhibitor celecoxib. The tumor regressed rapidly after the application of celecoxib. Within the 20-month follow-up period, the patient showed remarkable regression without any signs and symptoms. Our case report provides further evidence for the efficacy of celecoxib in AF with CTNNB1 gene mutations. To our knowledge, this is the first report of AF treated with celecoxib under the guidance of the genetic testing. However, further studies are required.

## Introduction

Aggressive fibromatosis (AF), also known as desmoid tumor, is aggressive, destructive, and infiltrative soft tissue neoplasms. It has a high risk of recurrence, but lacks the ability to metastasize. AF accounts for 3% of all soft-tissue malignancies with an incidence of 2.4–4.3 new patients per million population per year.^1^ Overall AF is present in people between 15–60 years old, and in more than twice as many women as men.^2^ There are two different types. The first type is mostly sporadic. However, at least 7.5% of AF is associated with the other type, familial adenomatous polyposis (FAP), equating to an 800-fold risk compared to the general population.^3,4^ AF is classified into intra-abdominal, extra-abdominal and abdominal wall according to sites. Extra-abdominal fibromatosis occurs in the limb, the head and neck or the trunk, while intra-abdominal fibromatosis involves the pelvis, retroperitoneum, mesentery and the intestinal wall.^5,6^ Historically, surgical resection was the first-line therapy for AF. However, depending on margins, recurrence rates of up to 27%-54% are reported.^7^ Recently, due to the spontaneous regression of AF without treatment and high recurrence rate, “watch and wait” policy has been proposed. Radiotherapy has been used either as an adjunct to surgery or in isolation. It improves local control rate regardless of negative or positive margins. The complications include tissue fibrosis and radiation-induced neoplasms, which are associated with total doses>56Gy.^8^ Several agents have been used successfully including hormone therapy, NSAIDs and tyrosine kinase inhibitors. Here, CTNNB1 gene mutations are present as shown in the report of the patient with chest wall AF. The tumor showed remarkable regression after celecoxib therapy.

## Case report

**Figure 1.**
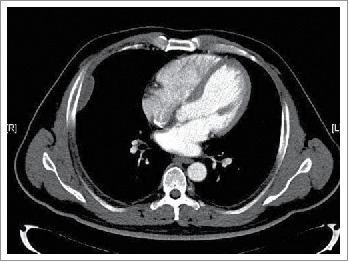
Tumor on the chest wall before surgery.

**Figure 2.**
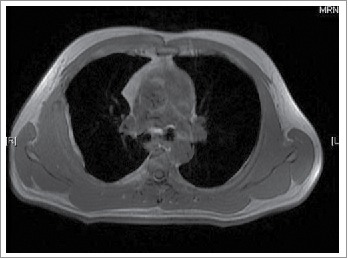
MRI demonstrates thickened soft tissue lesion four months after surgery.

**Figure 3.**
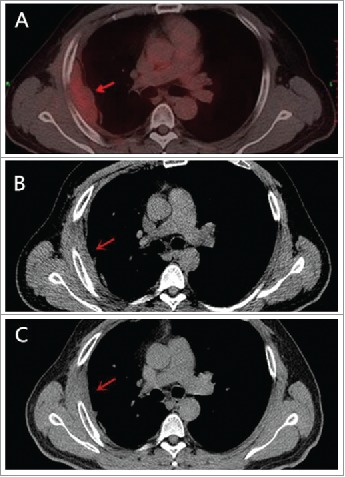
Tumor remarkable shrinkage was confirmed. PET-CT before the commencement of celecoxib on April 2014 A. CT scan on November 2015 B. and on October 2016 C. **s**howed that tumor was smaller after using celecoxib.

A 39-year-old man first presented to our outpatient clinic with chest wall pain in June 2014. Upon physical examination, an irregularly mass (approximately 5× 1 cm) was noted in the right rib. An enhanced computed tomography (CT) showed an approximately 46× 13 mm soft-tissue mass between the inside of the fifth and sixth rib on the right side, and the mass was enhanced slightly with periosteal reaction in adjacent ribs (Fig. 1). He neither has FAP nor any family history. Given the patient,s strong desire, the surgery was performed on July 16, 2014. During the operation, we detected that the lesion was an approximately 5× 5.5 cm soft-tissue mass, grey and toughening. It did not involve the rib but the surrounding skeletal muscles through macroscopical observation. Examination of frozen issue section found tumor cells, and the pathologic analysis revealed spindle-cellular tumors. The surgery was successful with no further treatment. CT or MRI was rechecked regularly after operation. Gradually, the patient felt pain on the chest wall once more. MRI showed obvious thickened soft tissue lesion on the chest wall with low signal intensity on T1 weighted images, and high signal intensity on T2 in November 2014 (Fig. 2). CT-guided biopsy indicated local recurrence.^18^ F-FDG PET/CT images showed the SUVmax of soft tissue on the chest wall was 4.5 on January 29, 2015. Tomotherapy had been given at doses of 60 Gy (2Gy/fraction) in February 2015. However, a follow-up ^18^F-FDG PET/CT demonstrated intense^18^ F-FDG uptake in a spindle soft tissue mass with an SUVmax of 4.3 on April 28, 2015 (Fig. 3A). For personalized therapy, genetic testing was performed in BGI on Jane 24, 2015. 508 cancer-related genes on tumor tissue DNA and peripheral blood DNA were detected through an Illumina Hiseq 2500 platform. The presence of a p.T41A mutations on the CTNNB1 gene was detected, which indicated his sensitivity to the celecoxib, the COX-2 inhibitor, based on targeted therapies and carboplatin-gemcitabine chemotherapy (Table 1). After a 5-month treatment with Celecoxib (200 mg/day), the CT scan showed tumor regression (Fig. 3B). Until now, the patient showed remarkable regression without any signs or symptoms.

**Table 1.** Result of gene mutation. CTNNB1 gene codes β-catenin, a kind of adhesive connections protein. ARID1B gene could code protein, playing an important role in cell cycle activation. The protein coded by SMC3 gene could be the kernel protein or secretory protein in specific cells. MSH5 gene codes mutS protein family, participating in DNA mismatch repair and meiotic recombination.

Gene	Base mutation	Amino acid mutation	Mutation frequency
CTNNB1	c.[121A>G]	p.[T41A]	4%
ARID1B	c.[811G>T]	p.[A271S]	2.04%
SMC3	c.[170G>A]	p.[R57H]	2.11%
MSH5	c.[136_138delGAG]	p.[E46del]	3.94%

## Discussion

Sporadic AF is characterized by somatic mutations in the β-catenin genes (CTNNB1) or adenomatous polyposis coli (APC) gene. Several studies demonstrate that around 85% of sporadic desmoid tumors harbor mutations in exon 3 of CTNNB1 gene, and three kinds of CTNNB1 status, 41A (59%), 45F (33%) and 45P(8%), are found. ^9^ Studies indicate the presence of mutation 45F obviously increases the risk of recurrence in comparison with the other CTNNB1 mutations or AF without 45F mutation.^10^ By contrast, FAP-associated AF is caused by germline APC mutations.^11–13^ Both CTNNB1 and APC are the parts of the WNT signaling pathway, which promotes the degradation of β-catenin. In the Wingless/Wnt signaling pathway, a protein named Dishevelled (Dvl) binds to the cell-surface receptor after Wnt binding. A multiprotein complex consisting of APC, glycogen synthase kinase (GSK) 3β and Axin is formed and then combined with β-catenin, which is phosphorylated by GSK-3β and then degraded by the proteasome. However, if mutated, either with the occurrence of CTNNB1 or APC, the multiprotein complex could not be generated, leading to β-catenin protein stabilisation. As a result, β-catenin accumulates in the cytoplasm and subsequently translocates to the nucleus, where it binds to transcription factors of the Tcf-LEF family, through which transcription of target genes including c-JUN, c-MYC, Nr-CAM, MMP7, cyclin D1 and COX-2 is triggered.^9,11,14^ Cyclooxygenase-2(COX-2) is an enzyme which could adjust the synthesis of prostaglandins and plays important roles in lots of biological processes such as immune function regulation. However, overexpression of COX-2 results in increasing the expression of platelet-derived growth factors (PDGF), which contributes to tumorigenesis by stimulating angiogenesis and invasiveness. In addition, COX-2 has also been found to promote apoptosis resistance e.g. by altering the relative levels of survivin and of pro- and anti-apoptotic proteins of the Bcl-2 family. COX-2 expression is elevated in several tumors, including AF. Celecoxib (Celebrex), an unique NSAIDs/coxibs, is able to potently induce anti-tumor responses by both COX-2 dependent and independent mechanisms. Celecoxib would interfere with prostaglandin(PG)-mediated up regulation of anti-apoptotic proteins by inhibiting COX-2. However, the pro-apoptotic effects do not critically rely on COX-2 inhibition.^15^ Celecoxib could induce cell death independently from COX-2 mainly by activation of an intrinsic, mitochondria-dependent apoptosis pathway. And it is suggested that celecoxib is able to suppress survivin expression, inhibit the activity of sarcoplasmic/endoplasmic reticulum calcium ATPase (SERCA), block the activity of cyclin-dependent kinases (CDKs), downregulate the activity of 3-phosphoinositide-dependent protein kinase-1 (PDK1), a critical upstream regulator of protein kinase B (PKB/Akt) and reduce beta-catenin signaling in the absence of COX-2.^16, 17^ In particular, the Wnt/β-catenin pathway is a classical pathway that has been suggested to be a COX-2-independent target for Celecoxib.^18,19^ It has been reported that Celecoxib could induce dephosphorylation of substrates for the RTKs c-Met, leading to decreased downstream signaling. The decrease in c-Met activation is accompanied with an increase in (GSK)3β kinase activity, resulting in a rapid increase in phosphorylation of β-catenin. Hence, celecoxib makes a decrease of TCF-β-catenin–dependent transcription although the biochemical basis for these effects has not been elucidated clearly.^20–22^

Considering the unpredictable natural history, the therapy remains enigmatic. The principle treatment has been considered for surgeries with or without radiation. Due to its biological behavior, therapeutic strategy has been shifted and watchful waiting may become an appropriate management.^23^ Studies indicate that AF expresses nuclear estrogen receptor-β, which provides a biological basis for hormone therapy, but the response rate to anti-hormonal therapies is low.^24^ Individualized therapy is required, the aims of which include reducing local recurrence rates and function loss. As genomic testing is widely available, the role of mutations has been studied, whereas agents that have CTNNB1 as a molecular target need to be further evaluated^2^. Targeted therapies including imatinib and Sorafenib (Tyrosine Kinase Inhibitors) have also been investigated. Prospective phase II clinical trial suggests that imatinib is active in AF,^25^ and a randomised, double-blind, placebo-controlled phase III clinical trial of Sorafenib is ongoing.

In the present case, local recurrence occurs repeatedly despite operation and radiotherapy. Therefore, the genomic analysis is utilized to direct clinicians in selection treatment drugs. The patient showed remarkable regression after the application of celecoxib according to genetic testing. At the end of 20-monthfollow-up, the patient showed no tumor deterioration and remained asymptomatic.

A Pilot Study reported that 19 (95%) of the 20 patients with Extra-Abdominal Desmoid Tumors had a final status of SD or got better with the use of Meloxicam, a Cyclooxygenase-2 inhibitor^26^. Yu-Chieh Wang et al reported that a patient who received celecoxib achieved complete remission.^27^ Another case that was successfully treated with etodolac, another COX-2 inhibitor, was previously reported by Tanaka et al.^28^ However, above cases used the COX-2 inhibitor without genetic testing.

In conclusion, as shown in our case report, an AF recurrence was successfully treated with celecoxib under the guidance of the genetic testing, which provides further evidence for NSAID. Multicenter randomized controlled trials are required in the future.
